# Increased brain volume in the early phase of aneurysmal subarachnoid hemorrhage leads to delayed cerebral ischemia

**DOI:** 10.3389/fsurg.2024.1467154

**Published:** 2024-09-19

**Authors:** Chao Sun, Bin Qin, Jingyu Zhang, Yidan Liang, Min Cui, Qiang Yang, Yanglingxi Wang, Jian Gong, Yi Xiang

**Affiliations:** ^1^ Department of Neurosurgery, Chongqing Emergency Medical Center (Chongqing University Central Hospital), Chongqing, China; ^2^ Department of Neurosurgery, The First Affiliated Hospital of Chongqing Medical University, Chongqing, China; ^3^ Department of Neurosurgery, Army Medical University (Third Military Medical University), Chongqing, China

**Keywords:** aneurysmal subarachnoid hemorrhage (aSAH), early brain injury (EBI), 3D-Slicer, swelling rate of brain volume, delayed cerebral ischemia (DCI)

## Abstract

**Objective:**

To investigate the correlation between the swelling rate of brain volume within the first 48 h after aneurysmal subarachnoid hemorrhage and the subsequent development of delayed cerebral ischemia.

**Methods:**

A retrospective analysis was conducted on patients with spontaneous aneurysmal subarachnoid hemorrhage admitted to the Neurosurgery Intensive Care Unit of the First Affiliated Hospital of Chongqing Medical University between January 2020 and January 2023. The clinical data, treatment outcomes, and imaging data were analyzed. Brain volume was evaluated using 3D-Slicer software at two time points post-hemorrhage: within the first 24 h and between 24 and 48 h. The swelling rate of brain volume was defined as the ratio of the absolute difference between two measurements to the smaller of values. Patients were categorized into two groups based on established diagnostic criteria of delayed cerebral ischemia. Univariate and multivariate logistic regression analyses were performed to identify factors influencing delayed cerebral ischemia.

**Results:**

A total of 140 patients were enrolled in this study. 46 patients experienced delayed cerebral ischemia after bleeding. The swelling rate of brain volume was larger in the DCI group (10.66 ± 8.45) compared to the non-DCI group (3.59 ± 2.62), which showed a statistically significant difference. Additionally, advanced age, smoking history, history of hypertension, loss of consciousness, poor Hunt-Hess grade, high mFisher score, brain volume within 24 h, and IVH were also statistically different between the two groups. Multivariate logistic regression analysis revealed that the swelling rate of brain volume was an independent risk factor for DCI with adjusting the advanced age, smoking history, history of hypertension, poor Hunt-Hess grade, high mFisher score, brain volume within 24 h, and IVH.

**Conclusion:**

Brain volume significantly increased in patients with aneurysmal subarachnoid hemorrhage during the early phase (within 48 h post-onset). The larger swelling rate of brain volume is an independent risk factor for the development of delayed cerebral ischemia, and it may hold significant predictive value for the incidence of delayed cerebral ischemia.

## Backgroup

Aneurysmal subarachnoid hemorrhage (aSAH) is a catastrophic cerebrovascular disease with high mortality and morbidity (1). Despite continuous improvement and innovation of diagnostic methods and treatment techniques, the introduction of microsurgical operative techniques especially (2), which led to steady reduce the mortality from aSAH recently, the mortality of aSAH is still high (3). Even if it survives, there will be residual sequelae with a high disability rate. Multiple factors determine the clinical outcomes of patients, including the volume of the bleeding, patients' initial clinical signs and symptoms, whether early re-bleeding occurs or not, and the presence or absence of delayed cerebral ischemia (DCI) (4). DCI is one of the more serious complications of aSAH (5), which usually occurs within 14 days after bleeding (6). Ruptured intracranial aneurysms leads to destruction and decomposition of red blood cells, which stimulate the production of vasoconstrictor substances such as endothelin, so that the cerebral vessel will contract strongly and develop cerebral vasospasm (CVS). And CVS is a considerable factor leading to DCI, but it is not the only determinant (7). The current gold standard in diagnosis of DCI is digital subtraction angiography (DSA), the mFisher score (mFS) is to predict the vasospasm and DCI after onset by evaluating the amount and location of hemorrhage on brain CT (8, 9). The mFS score relies mainly upon the clinical diagnosis of the neurosurgeon. However, as we have learned from recent studies, DCI itself is a complex multifactorial process that cannot be explained completely by the amount of blood in the cisternal space and large vessel vasospasm (10). Appropriately, there has been increasing attention focused on early brain injury (EBI) (11). It refers to the direct injury of brain tissue within 72 h after bleeding, including a series of pathophysiological changes in brain tissue. Brain swelling is a radiological imaging manifestation of EBI, which is associated with DCI (12). Consequently, the lack of dependable quantification metrics for assessing severity of brain swelling impedes further evaluation and inquiry into EBI. The efficacy of quantitative imaging is contingent upon sophisticated analytical software and methodologies. 3D-Slicer, a volumetric analysis software, is extensively utilized in neurosurgery for processing, analyzing, and visualizing diverse medical neuroimaging datasets through its specialized modules (13). In this study, we assessed the brain volume (BV) and the swelling rate of brain volume (SRBV) utilizing 3D-Slicer, which is predicated on threshold segmentation techniques. We propose the hypothesis that the SRBV measured during the acute phase of aSAH serves as a numerical indicator of the severity of EBI. Furthermore, SRBV could potentially be employed as an alternative clinical monitoring indicators based on CT, which may have the capacity to predict the occurrence of DCI in patients with aSAH.

## Subjects and methods

### Participants

Patients diagnosed with aSAH and admitted to the Neurosurgery Intensive Care Unit (NSICU) at the First Affiliated Hospital of Chongqing Medical University between January 2020 and January 2023 were documented. This research obtained approval from the Institutional Review Board (IRB) of the First Affiliated Hospital of Chongqing Medical University, adhering to the ethical standards set forth in the Declaration of Helsinki. Given the retrospective design of this investigation, informed consent was not required. The diagnosis of SAH was established by the admission CT scan. Patients with aSAH and a CT within 24 h of ictus were included.

For this study, the exclusion criteria included the following: patients with SAH due to trauma, arteriovenous malformation rupture, vasculitis; patient with previous history of brain injury and related chronic disease; patients with secondary brain swelling caused by cerebral ischemia, rebleeding, intracranial infection, and other complications; patients who undergo surgery within 48 h; and patients with the estimated survival time was <1 year.

### Clinical variables

We recorded baseline demographic data (age and gender), previous medical history (hypertension, hyperlipidemia, and diabetes), social history, and rating scales on admission [Hunt-Hess grade (HH), World Federation of Neurosurgical Societies (WFNS), modified Fisher scale (mFS)]. Poor clinical condition was defined as high WFNS (4–5) and HH (4–5). Large amount of bleeding was defined as high mFS (3–4). The neurological and general medical assessments of all subjects were conducted by two neurologists upon admission. Treatment protocols for all patients were implemented in accordance with the existing clinical guidelines (1).

### Radiographic variables

DCI (14) was defined as the deterioration of clinical symptoms, including new focal neurological deficits (e.g., hemiplegia, aphasia, apraxia, hemianopsia or neglect), or decreasing in consciousness (at least 2 points of GCS score, including total score or single score), which lasts for at least 1 h and does not occur in immediately after operation, or reexamination of head CT revealed a new infarct focus, which did not appear in the first admission or reexamination of head CT after surgery. Secondary neurological deterioration is not attributed to other causes (i.e., including fever, infection complications, hydrocephalus, epilepsy, respiratory failure or electrolyte disorder) after appropriate clinical, imaging, and laboratory evaluation. The diagnosis of DCI was performed by the physician in charge and the neurological function monitoring and evaluation team. In addition, two senior neurosurgeons confirmed the final diagnosis of DCI through the review and evaluation of the patient's case data. Evidence of vasospasm in the great artery has been used to further support the diagnosis of DCI, but is not a necessary condition for diagnosis. Finally, aSAH patients included in this study were divided into DCI group and non-DCI group for statistical analysis.

### Image data processing

Brain CT scans were quantified using 3D-Slicer version 4.10.2, and measurements were carried out based on the threshold algorithm. The methodology that was mentioned in the previous article serves as the foundation for this study (15). In the assessment of BV, avoiding the deviation caused by human factors, we adopted the following means: two neurosurgeons independently calculated the BV of every patient, and the final result of BV was the average value obtained by two values. And then, BV was evaluated at two time points post-hemorrhage: within the first 24 h (1stBV) and between 24 and 48 h (2ndBV). The change in brain volume (CIBV) is the absolute difference between the two measurements (|2ndBV-1stBV|). The SRBV was defined as the ratio of the absolute difference between two measurements to the smaller of those two values [CIBV/1stBV]. The quantitative measurements for intraventricular hemorrhage (IVH) and ambient cistern blood was also based on threshold algorithm. The CT value of hemorrhagic area is higher than that of brain tissue within early phase of aSAH. Firstly, we use tools to mark the target region by operating watershed module. Secondly, we can separated the hemorrhagic area from brain tissue by operating segmentation module. Ultimately, quantifying IVH and ambient cistern blood in volume measurement module.

### Statistical analysis

In this study, participants were categorized into two cohorts based on the presence or absence of DCI. The selection of statistical tests for continuous variables was predicated on their distribution; normally distributed variables were compared using the unpaired Student's *t*-test, while non-normally distributed variables were assessed with the Mann-Whitney *U*-test. For categorical variables, the Fisher's exact test was employed for small sample sizes, and the chi-squared (χ^2^) test was utilized for larger datasets. Significant variables were identified through univariate analysis with a threshold of *p* < 0.05 and subsequently integrated into multivariate logistic regression models to ascertain the independent risk factors for DCI. The outcomes of this analysis are presented as odds ratios (OR) with their corresponding 95% confidence intervals (CI) and *p*-values. Data analysis was conducted utilizing SPSS software, version 25.0, with statistical significance defined as *p* < 0.05.

## Result

### Baseline characteristics

In our research, the data for 227 patients were recorded, but only 140 patients met the inclusion criteria and were included in the analysis. 40 patients were over 60 years old (28.57%). 101 patients (72.14%) were female, and 39 patients (27.86%) were male. 29 patients (20.71%) had a history of smoking before onset of illness, and 60 patients (42.86%) have a history of hypertension. The HH scores of the 9 patients (6.43%) upon admission was poor level (4–5), 17 patients (12.14%) had a poor score (4–5) of WFNS at admission. In terms of radiological imaging, 56 patients (40.00%) were assessed as poor mFS scale (3–4). In 9 patients (6.43%), their aneurysms exceeding 10 millimeters were identified, the majority of patients (87cases, 62.14%) with aneurysms have them located in the anterior circulation, and 29 patients (20.71%) have multiple aneurysms. The mean of 2ndBV was larger than the 1stBV [1,180.49 ml (±106.88) vs. 1,118.03 ml (±112.46), *p* < 0.05]. The mean CIBV and SRBV were 62.47 ml (±59.83) and 5.86% (±6.05), respectively (Table 1).

**Table 1:** Study patient characteristics.

	*n* = 140
Baseline data *N* (%)	
Age (>60 years)	40 (28.57)
Female	101 (72.14)
Smoking	29 (20.71)
Alcohol	28 (20.00)
Hypertension	60 (42.86)
Hyperlipidemia	26 (18.57)
Diabetes	9 (6.43)
Clinical data *N* (%)	
LOC	29 (20.71)
WFNS 4–5	17 (12.14)
HH 4–5	9 (6.43)
Radiological data *N* (%)	
mFS 3–4	56 (40.00)
Aneurysm *N* (%)	
Size of aneurysm (>10 mm)	9 (6.43)
Set of aneurysm (ACA)	87 (62.14)
Multiple aneurysms	29 (20.71)
Volume in ml, mean (SD)	
2ndBV	1,180.49 ± 106.88
1stBV	1,118.03 ± 112.46
CIBV	62.47 ± 59.83
SRBV	5.86 ± 6.05
Ambient cistern blood	1.48 ± 3.82
IVH	5.57 ± 3.82

Bold value indicates *p* < 0.05, LOC, loss of consciousness at ictus; BV, brain volume; 1stBV, the brain volume within 24 h after bleeding; 2ndBV, the brain volume in the early course (within 24 h–48 h) of aSAH; CIBV, the change in brain volume (|2ndBV-1stBV|); SRBV, the swelling rate of brain volume; DCI, delayed cerebral ischemia; HH, Hunt-Hess scale; mFS, modified Fisher scale; WFNS, World Federation of Neurosurgical Societies; ACA, anterior circulation aneurysm; IVH, intraventricular hemorrhage.

### SRBV and DCI

Compared to the non-DCI group, the DCI group has a greater proportion of elderly patients (22.34% vs. 41.30%, *p* < 0.05). 27 patients (58.70%) in the DCI group had a long history of hypertension, whereas 33 patients (35.11%) in the non-DCI group (*p* < 0.05). There was a significant difference between the groups in the loss of consciousness (LOC) and poor HH grade (*p* < 0.05), and Patients with SAH who have LOC and/or poor admission scores seem to be more prone to developing DCI. The mean of SRBV in the two groups (DCI vs. non-DCI) was normally distributed, with average values (±SD) of 10.66% (±8.45) and 3.59% (±2.62), which was a significant difference between the two groups (*p* < 0.05). Additional baseline characteristic details are shown in Table 2.

**Table 2:** Univariate analysis of characteristics of SAH patients (*n* = 140).

Variables	Non-DCI (−) (*N* = 94)	DCI (+) (*N* = 46)	*P*-value
Baseline data *N* (%)			
Age (>60 years)	21 (22.34)	19 (41.30)	**0** **.** **020**
Female	65 (69.15)	36 (78.26)	0.259
Smoking	15 (15.96)	14 (30.43)	**0** **.** **047**
Alcohol	16 (17.02)	12 (26.09)	0.208
Hypertension	33 (35.11)	27 (58.70)	**0** **.** **008**
Hyperlipidemia	18 (19.15)	8 (17.39)	0.802
Diabetes	7 (7.45)	2 (4.35)	0.718
Clinical data *N* (%)			
LOC	13 (13.83)	16 (34.78)	**0** **.** **004**
WFNS (4–5)	8 (8.51)	9 (19.57)	0.060
HH (4–5)	1 (1.06)	8 (17.39)	**0** **.** **001**
Radiological data *N* (%)			
mFS (3–4)	24 (25.53)	32 (69.57)	**<0** **.** **001**
Aneurysm *N* (%)			
Size of aneurysm (>10 mm)	4 (4.26)	5 (10.87)	0.154
Set of aneurysm (ACA)	56 (59.57)	31 (67.39)	0.664
Multiple aneurysms	20 (21.28)	9 (19.57)	0.814
Volume in ml, mean (SD)			
2ndBV	1,173.76 ± 105.85	1,193.62 ± 108.68	0.303
1stBV	1,133.54 ± 105.08	1,086.96 ± 121.99	**0** **.** **021**
SRBV	3.59 ± 2.62	10.66 ± 8.45	**<0** **.** **001**
Ambient cistern blood	1.37 ± 2.01	1.71 ± 2.33	0.385
IVH	4.86 ± 3.30	7.02 ± 4.41	**0** **.** **005**

Bold value indicates *p* < 0.05; LOC, loss of consciousness at ictus; BV, brain volume; 1stBV, the brain volume within 24 h after bleeding; 2ndBV, the brain volume in the early course (within 24 h–48 h) of aSAH; CIBV, the change in brain volume (|2ndBV-1stBV|); SRBV, the swelling rate of brain volume; DCI, delayed cerebral ischemia; HH, Hunt-Hess scale; mFS, modified Fisher scale; WFNS, World Federation of Neurosurgical Societies; ACA, anterior circulation aneurysm; IVH, intraventricular hemorrhage.

### SRBV and clinical score

The analysis showed that there were significant differences in SRBV between poor score group (mFS 3–4) and good score group (mFS 1–2) (Table 3). Similar results were found in the statistical analysis of HH grade (Supplementary Table S1) and WFNS grade (Supplementary Table S2). The higher the score of the clinical admission scale, the larger the SRBV.

**Table 3:** Univariate analysis of varying degree of clinical score.

Volume in ml mean (SD)	mFS 1–2	mFS 3–4	*P*
2ndBV	1,167.86 ± 104.10	1,198.92 ± 109.02	0.092
1stBV	1,122.41 ± 112.52	1,111.96 ± 113.54	0.592
SRBV	4.47 ± 4.80	8.08 ± 7.44	**0** **.** **002**
Ambient cistern blood	1.20 ± 1.85	1.91 ± 2.42	0.065
IVH	4.44 ± 3.11	7.27 ± 4.18	**0** **.** **001**

Bold value indicates *p* < 0.05; LOC, loss of consciousness at ictus; BV, brain volume; 1stBV, the brain volume within 24 h after bleeding; 2ndBV, the brain volume in the early course (within 24 h–48 h) of aSAH; CIBV, the change in brain volume (|2ndBV-1stBV|); SRBV, the swelling rate of brain volume; DCI, delayed cerebral ischemia; HH, Hunt-Hess scale; mFS, modified Fisher scale; WFNS, World Federation of Neurosurgical Societies; ACA, anterior circulation aneurysm; IVH, intraventricular hemorrhage.

### Independent risk factors for DCI

46 of the 140 (32.86%) subjects were in the DCI group, and 94 (67.14%) were in the non-DCI group. This is in line with the previously reported incidence of DCI after SAH (16). Multivariate logistic regression analyses indicated that smoking history, poor mFS scale (3–4), and SRBV were independent predictors of DCI. The model 3 showed that for every 1% increase in the SRBV, the odds of occurring DCI increased by a factor of 1.281 (95% CI = 1.121 ∼ 1.464) after adjusting for advanced age, smoking history, hypertension, poor HH grade (4∼5), high mFisher score, 1stBV, IVH. Additional details are shown in Table 4.

**Table 4:** Unadjusted and adjusted odds ratios for SRBV to predict DCI.

Clinical variables	Model 1			Model 2			Model 3		
	OR	95% CI	*p*-value	OR	95% CI	*p*-value	OR	95% CI	*p*-value
SRBV, per 1% increase	1.297	1.161–1.450	<0.001	1.332	1.176–1.509	<0.001	1.281	1.121–1.464	<0.001

Bold value indicates *p* < 0.05, OR, odds ratio; CI, confidence interval; LOC, loss of consciousness at ictus; BV, brain volume; 1stBV, the brain volume within 24 h after bleeding; 2ndBV, the brain volume in the early course (within 24 h–48 h) of aSAH; CIBV, the change in brain volume (|2ndBV-1stBV|); SRBV, the swelling rate of brain volume; DCI, delayed cerebral ischemia; HH, Hunt-Hess scale; mFS, modified Fisher scale; IVH, intraventricular hemorrhage.

Significant variables in the univariate analysis (*P* < 0.05) were included in a multivariate logistic regression analysis.

Model 1: unadjusted.

Model 2: adjusted for smoking and LOC.

Model 3: adjusted for advanced age, smoking, hypertension, poor HH grade (4–5), high mFS grade (3–4), 1stBV, IVH.

## Discussion

Our main finding is that the SRBV, numerical indicator of the severity of EBI, is an independent predictor of DCI. Two interesting results are recognized: (1) comparing with DCI group, the SRBV in the Non-DCI group was smaller, and (2) patients with a larger SRBV are more likely occurred DCI. Based on the above results, brain swelling after aSAH is a crucial target for timely treatment, and the SRBV may be an alternative marker for therapeutic efficacy in aSAH treatment.

Recently, there has been controversy over using CVS as a major factor to explain the occurrence of DCI after bleeding (17). Research has shown that there is not necessarily a correlation between the distribution of cerebral ischemic regions and spasmodic arteries, and brain regions without vasomotor can also develop DCI/hypoperfusion (18–20). Trials evidence has shown that preventing vasospasm does not necessarily reduce all-cause mortality or DCI, laying the foundation for changing our understanding of the pathology of DCI (21). More studies have shown that the occurrence of DCI is a comprehensive change of multiple physiological and pathological processes (22). This includes EBI, formation of microthrombus, cortical depolarization, etc. (11, 23, 24). Increasingly attention is turning to the research of EBI, which starts within the first few minutes to several hours after the rupture of the aneurysm and lasts for several days (25). The concept of EBI is important because it reflects the obvious clinical situation, especially the initial clinical manifestations, which are the most significant prognostic indicators, whether measured by HH or WFNS. There are reports that brain swelling is an imaging manifestation for EBI (26), and the binary qualitative diagnosis of the presence or absence of brain swelling in clinical practice limits its wider practicality and deeper research. Brain swelling should be constantly changing with the physiological instability of the disease after onset. Sung-Ho's research demonstrates that there may be an increase in brain volume during EBI, which is related to the occurrence of DCI (27). Our study found that compared to non-DCI group [3.59% (±2.62)], DCI group of the value of SRBV [10.66% (±8.45)] was larger (*p* < 0.05). SRBV might can serve as a visual indicator of EBI to predict the occurrence of DCI after bleeding, which may help in multimodal monitoring of patients. Similar to the pathophysiology of brain swelling, the changes of BV may reflect tissue and microvascular damage caused by sudden cessation of intracranial circulation after aneurysm rupture and bleeding (28). Insufficient cerebral perfusion caused by the surge in intracranial pressure can lead to transient diffuse ischemic encephalopathy (29). In Table 3, SRBV in aSAH patients within 48 h after onset showed significant differences (*P* < 0.05) between severity levels of classical clinical score. The worse clinical score of the patient on admission, the more significant the increase in SRBV. Patients with poor WFNS grading at admission are prone to excessive intracranial pressure, which exacerbates factors of microvascular and cell damage, leading to greater changes in SRBV.

A confounding factor that needs to be carefully considered is the age of the patient. The brain undergoes physiological atrophy with age, which has been validated by a large amount of experimental data (30). In order to eliminate this inevitable factor as much as possible, we intentionally made the target value into a relative value. In our research, there was differences in advanced age between two groups (DCI vs. non-DCI) in univariate analysis. However, after incorporating multiple factor analysis, consistent with recent study (31), which indicate that advanced age is not an independent risk factor of DCI. There is currently controversy over research on predicting the occurrence of DCI in patients after bleeding based on age. Some studies show that the older the age, the lower the incidence rate of DCI (32, 33). However, another study found the opposite result, older patients, compared with the younger, are more likely to suffer from DCI (34). Apart from the two completely opposite results mentioned above, some studies have found that age may not have an impact on the occurrence of DCI (35, 36). This difference can be attributed to the differences in age group division in each study. Advanced age patients have a larger subarachnoid space and a greater volume of blood clots after bleeding, which is considered to be the cause of DCI (37). On the contrary, another hypothesis is that the cerebral vessels of elderly patients are stiffer (caused by atherosclerosis, increased collagen fibers, etc.), which makes them more resistant to vasospasm (31).

In this study, we found that the mean of 2ndBV is larger than 1stBV, which has statistical significance. It may indicate that there is indeed a varying degree of numerical increase in BV within the acute phase of aSAH, which is consistent with previous report (12). We found that the 2ndBV was not significantly different between the two groups (DCI vs. non-DCI), but the SRBV demonstrated completely opposite results. This may be related to the physiological anatomy of the brain. So, Focusing exclusively on BV at a single time point post-onset, while disregarding its dynamic alterations throughout the disease progression, could yield biased outcomes. Potentially detrimental post-hemorrhagic pathophysiological changes may manifest as an enlargement of BV, potentially resulting in the ineffectiveness of conservative management and necessitating decompressive hemicraniectomy (DHC). However, the efficacy and appropriate indications for DHC remain subjects of debate (38). The mFS scale is a classical radiological grading system utilized to predict the occurrence of vasospasms after bleeding. It accomplishes this by evaluating the degrees of hemorrhage within the subarachnoid space and the lateral ventricles (8). The outcomes of this study are consistent with our prediction that there are differences between two groups [(mFS 1–2) vs. (mFS 3–4)] in variable of blood in lateral ventricle. The higher the score, the greater the volume of blood observed within the lateral ventricle. Quantitative analysis of IVH standardizes assessment of SAH, which will not be affected by physician subjectivity. Furthermore, this methodology is also applicable for assessing cases of unilateral ventricular hemorrhage, a capability not offered by the mFS scale. Our findings showed that other poor clinical scores were related to the larger SRBV. This may be attributed to the disruption of brain tissue in the early stage after SAH, which may be at the level of microcirculation (39). The SRBV may reflect additional changes in brain physiology that represent microcirculation changes.

IVH is common complication following aSAH, and the correlation between IVH and DCI has been a research focus. It is consistent with previous report (40) that IVH is not an independent risk factor for DCI in our research. The primary driver of DCI may be associate with ambient cistern blood, and the damaging mechanism of IVH may not be through DCI but alternative neuronal injury or inflammatory pathways (41). Others hold quite the opposite view, they suggest that IVH is predictive factor for DCI. The etiology potentially lies in the obstruction of cerebrospinal fluid flow by blood, which leads to elevated intracranial pressure and consequently impairs cerebral blood flow, thereby augmenting the risk of DCI occurrence (42). Another study highlights that DCI is a multifactorial process that is not only related to IVH, but also involves a variety of pathological factors such as microcirculation dysfunction, damage to the glial lymphatic system, inflammation, and electrical disturbance of nerves (7). The relationship between IVH and DCI may be influenced by individual differences, bleeding severity, treatment measures, and other factors. Therefore, multiple factors need to be considered comprehensively when evaluating IVH as a risk factor for DCI. In addition to IVH, patients may also develop subarachnoid blood, intraparenchymal blood, and ambient cistern blood. However, researches showed that subarachnoid blood (43) and intraparenchymal blood (44) lack potential ability to predict DCI compared to IVH and ambient cistern blood. Consequently, we have abandoned the calculation of these values in study.

Although potential confounding factors were accounted for, it is essential to acknowledge the limitations inherent in our research. Firstly, our research is constrained by the limitations of a single-observation study design. Nonetheless, we mitigated potential bias by ensuring that imaging operators were blinded to patients' clinical details during the imaging assessments. Secondly, MRI provides a more precise assessment of BV compared to CT. However, MRI's accessibility is limited for patients with severe aSAH. However, the majority of aSAH patients receive multiple CT scans while hospitalized. Finally, the processing of image processing is a semi-automatic method, and operation is time-consuming.

## Conclusion

In conclusion, BV increases in patients with aSAH during the early phase (within 48 h post-onset). A significant difference in the SRBV was observed between two groups (DCI vs. non-DCI), SRBV in DCI group is larger. The SRBV was quantified using semi-automated methods, which can be used as a volumetric representation of the severity of brain swelling. The SRBV may serve as a surrogate numerical indicator of EBI during the initial phase of onset, which can predict the occurrence of DCI. A larger SRBV in the early stage of aSAH was related to occurrence of DCI compared with the lower SRBV.

## Data Availability

The raw data supporting the conclusions of this article will be made available by the authors, without undue reservation.
