# Supplementary material for: Increased brain volume in the early phase of aneurysmal subarachnoid hemorrhage leads to delayed cerebral ischemia
Source: Front Surg. 2024 Sep 19;11:1467154. doi: 10.3389/fsurg.2024.1467154 (PMC11446905; doi:10.3389/fsurg.2024.1467154)
Supplement: Supplementary file 1 [file Table1.docx]

| **Supplementary Table 1 [Univariate Analysis of Varying Degree of Clinical Score](#结果表2)** | | | |
| --- | --- | --- | --- |
| **Volume in mL**  **mean (SD)** | **WFNS 0 ~ 3** | **WFNS 4 ~ 5** | **P** |
| 2^nd^BV | 1176.67 ± 105.51 | 1206.26 ± 115.77 | 0.286 |
| 1^st^BV | 1120.83 ± 113.70 | 1099.42 ± 105.98 | 0.464 |
| SRBV | 5.41 ± 5.93 | 9.60 ± 7.24 | **0.009** |
| Ambient cistern blood | 1.39 ± 2.03 | 2.14 ± 2.62 | 0.276 |
| IVH | 5.30 ± 3.85 | 7.55 ± 3.05 | **0.011** |
| ***Bold value*** indicates p < 0.05; ***LOC*** loss of consciousness at ictus, ***BV*** brain volume, ***1^st^BV*** the brain volume within 24 hours after bleeding, ***2^nd^BV*** the the brain volume in the early course (within 24h ~ 48h) of aSAH, ***CIBV*** the change in brain volume (\|2^nd^BV-1^st^BV\|), ***SRBV*** the swelling rate of brain volume, ***DCI*** delayed cerebral ischemia, ***HH*** Hunt-Hess scale, ***mFS*** modified Fisher Scale, ***WFNS*** World Federation of Neurosurgical Societies, ***ACA*** anterior circulation aneurysm, ***IVH*** Intraventricular hemorrhage | | | |

| **Supplementary Table 2** [**Univariate Analysis of Varying Degree of Clinical Score**](#结果表2) | | | |
| --- | --- | --- | --- |
| **Volume in mL**  **mean (SD)** | **HH 1 ~ 3** | **HH 4 ~ 5** | **P** |
| 2^nd^BV | 1175.23 ± 106.42 | 1253.95 ± 87.51 | 0.32 |
| 1^st^BV | 1122.30 ± 113.17 | 1059.00 ± 90.07 | 0.103 |
| SRBV | 4.91 ± 4.54 | 10.53 ± 9.06 | **0.001** |
| Ambient cistern blood | 1.37 ± 2.05 | 3.11 ± 2.44 | 0.067 |
| IVH | 5.48 ± 3.80 | 6.82 ± 4.20 | 0.312 |
| ***Bold value*** indicates p < 0.05; ***LOC*** loss of consciousness at ictus, ***BV*** brain volume, ***1^st^BV*** the brain volume within 24 hours after bleeding, ***2^nd^BV*** the the brain volume in the early course (within 24h ~ 48h) of aSAH, ***CIBV*** the change in brain volume (\|2^nd^BV-1^st^BV\|), ***SRBV*** the swelling rate of brain volume, ***DCI*** delayed cerebral ischemia, ***HH*** Hunt-Hess scale, ***mFS*** modified Fisher Scale, ***WFNS*** World Federation of Neurosurgical Societies, ***ACA*** anterior circulation aneurysm, ***IVH*** Intraventricular hemorrhage   \| **Supplementary Table 3 The Spearman-Correlation Analysis between SRBV and Covariates** \| \| \| \| --- \| --- \| --- \| \| **Clinical variables** \| **SRBV** \| \| \| **ρ** \| **P** \| \| SRBV \| 1 \|  \| \| Age>60 \| 0.187 \| **0.027** \| \| Smoke \| -0.008 \| 0.925 \| \| hypertension \| 0.17 \| **0.044** \| \| LOC \| 0.125 \| 0.143 \| \| HH (4~5) \| 0.349 \| **<0.001** \| \| mFS (3~4) \| 0.313 \| **<0.001** \| \| IVH \| 0.288 \| **<0.001** \| \| 1^st^BV \| -0.368 \| **<0.001** \| \| ***Bold value*** indicates p < 0.05; ***LOC*** loss of consciousness at ictus, ***BV*** brain volume, ***1^st^BV*** the brain volume within 24 hours after bleeding, ***2^nd^BV*** the the brain volume in the early course (within 24h ~ 48h) of aSAH, ***CIBV*** the change in brain volume (\|2^nd^BV-1^st^BV\|), ***SRBV*** the swelling rate of brain volume, ***DCI*** delayed cerebral ischemia, ***HH*** Hunt-Hess scale, ***mFS*** modified Fisher Scale, ***WFNS*** World Federation of Neurosurgical Societies, ***ACA*** anterior circulation aneurysm, ***IVH*** Intraventricular hemorrhage \| \| \| | | | |

| **Supplementary Table 4 Multicollinearity Test^a^** | | | |
| --- | --- | --- | --- |
| **Model** | | **Collinearity Statistics** | |
|  |  | **Tolerance** | **VIF** |
| 1 | SRBV | 1 | 1 |
| 2 | SRBV | 0.919 | 1.089 |
|  | mFS | 0.919 | 1.089 |
| 3 | SRBV | 0.918 | 1.089 |
|  | mFS | 0.918 | 1.089 |
|  | Smoke | 0.999 | 1.001 |
| 4 | SRBV | 0.905 | 1.105 |
|  | mFS | 0.911 | 1.098 |
|  | Smoke | 0.938 | 1.066 |
|  | >60y | 0.911 | 1.097 |
| 5 | SRBV | 0.876 | 1.141 |
|  | mFS | 0.899 | 1.113 |
|  | Smoke | 0.933 | 1.071 |
|  | >60y | 0.901 | 1.11 |
|  | LOC | 0.935 | 1.069 |
| **a** Dependent Variable: DCI, **VIF** variance inflation factor, ***LOC*** loss of consciousness at ictus, ***SRBV*** the swelling rate of brain volume, ***DCI*** delayed cerebral ischemia, ***HH*** Hunt-Hess scale, ***mFS*** modified Fisher Scale. | | | |
